# Mechanism of Immunopotentiation and Safety of Aluminum Adjuvants

**DOI:** 10.3389/fimmu.2012.00406

**Published:** 2013-01-10

**Authors:** Harm HogenEsch

**Affiliations:** ^1^Department of Comparative Pathobiology, College of Veterinary Medicine, Purdue UniversityWest Lafayette, IN, USA

**Keywords:** adjuvants, aluminum compounds, aluminum hydroxide, inflammasomes, inflammation, dendritic cells

## Abstract

Aluminum-containing adjuvants are widely used in preventive vaccines against infectious diseases and in preparations for allergy immunotherapy. The mechanism by which they enhance the immune response remains poorly understood. Aluminum adjuvants selectively stimulate a Th2 immune response upon injection of mice and a mixed response in human beings. They support activation of CD8 T cells, but these cells do not undergo terminal differentiation to cytotoxic T cells. Adsorption of antigens to aluminum adjuvants enhances the immune response by facilitating phagocytosis and slowing the diffusion of antigens from the injection site which allows time for inflammatory cells to accumulate. The adsorptive strength is important as high affinity interactions interfere with the immune response. Adsorption can also affect the physical and chemical stability of antigens. Aluminum adjuvants activate dendritic cells via direct and indirect mechanisms. Phagocytosis of aluminum adjuvants followed by disruption of the phagolysosome activates NLRP3-inflammasomes resulting in the release of active IL-1β and IL-18. Aluminum adjuvants also activate dendritic cells by binding to membrane lipid rafts. Injection of aluminum-adjuvanted vaccines causes the release of uric acid, DNA, and ATP from damaged cells which in turn activate dendritic cells. The use of aluminum adjuvant is limited by weak stimulation of cell-mediated immunity. This can be enhanced by addition of other immunomodulatory molecules. Adsorption of these molecules is determined by the same mechanisms that control adsorption of antigens and can affect the efficacy of such combination adjuvants. The widespread use of aluminum adjuvants can be attributed in part to the excellent safety record based on a 70-year history of use. They cause local inflammation at the injection site, but also reduce the severity of systemic and local reactions by binding biologically active molecules in vaccines.

## Introduction

Adjuvants are substances added to vaccines to enhance and direct the immune response. They are often necessary for the induction of a protective immune response against recombinant subunit antigens and protein toxins. The immunostimulatory effect of aluminum-containing adjuvants was first described in 1926 (Glenny et al., 1926). The investigators demonstrated that injection of diphtheria toxoid precipitated with alum (potassium aluminum sulfate) induced a stronger antibody response than soluble toxoid in guinea pigs. Subsequent studies showed that alum-precipitated diphtheria toxoid and tetanus toxoid also enhance protective immune responses in people (Jones, 1936; Volk and Bunney, 1942). Aluminum adjuvants are currently used in several human vaccines against infectious diseases, including vaccines against diphtheria, tetanus, pertussis, hepatitis B, anthrax, and diseases caused by *Haemophilus influenzae* and human papilloma virus (Baylor et al., 2002; Frazer et al., 2011). They are also used in immunotherapy for allergic diseases (Francis and Durham, 2004; Eifan et al., 2011), and are being evaluated for immunotherapy against autoimmune diabetes mellitus (Wherrett et al., 2011). Certain veterinary vaccines for protection of food animals and companion animals from infectious diseases are formulated with aluminum adjuvants (Lindblad, 2004).

Vaccines containing aluminum adjuvants induce an effective immune response that is primarily antibody-mediated. In spite of their longstanding use, the mechanism by which aluminum adjuvants selectively enhance the immune response is poorly understood. It is now generally accepted that the innate immune system plays a critical role in initiating and directing the adaptive immune response. Adjuvants enhance the adaptive immune response by activation of innate immune cells that in turn provides signals for activation of lymphocytes. Studies over the past decade have shed new light on the interaction of aluminum adjuvants with antigen-presenting cells and inflammatory cells, both of which appear to be critical in inducing and shaping the immune response. The possible mechanisms by which aluminum adjuvants enhance the immune response have been subject of several recent reviews (Aimanianda et al., 2009; Marrack et al., 2009; Kool et al., 2012). I will review the physical and chemical characteristics of aluminum adjuvants and their interaction with antigens and other immunomodulatory molecules, and discuss this along with the mechanism of immunostimulation. Aluminum adjuvants owe their popularity in part to their relatively low cost and long standing safety record (Lindblad, 2004). This review will conclude with a brief discussion of the safety of aluminum adjuvants.

## What are Aluminum-Containing Adjuvants?

Aluminum-containing adjuvants are often simply referred to as “alum.” This term should be avoided for two reasons. First, alum is the name of a specific chemical compound, hydrated potassium aluminum sulfate, KAl(SO_4_)_2_·12 H_2_O. Precipitation of a solution of alum and antigen was originally used for the preparation of aluminum-adjuvanted vaccines. The chemical composition of the aluminum precipitate depends on the type of ions present in the antigen solution. The precipitation method is difficult to reproduce in a consistent manner and has largely been replaced by adsorption of antigens to aluminum-containing gels. The second reason to avoid the term alum is that it fails to specify which type of aluminum-containing adjuvant was used for the vaccine preparation. The two main types of aluminum adjuvants that are commercially available are aluminum hydroxide adjuvant (AH) and aluminum phosphate adjuvant (AP). The physical and chemical composition of AH and AP are quite different and this has important implications for the formulation with antigens (Shirodkar et al., 1990; Lindblad, 2004; Hem and HogenEsch, 2007). AH is chemically aluminum oxyhydroxide, Al(O)OH, rather than Al(OH)_3_. It has a crystalline structure and is composed of primary needle-like nanoparticles that are 4.5 nm × 2.2 nm × 10 nm in size (Johnston et al., 2002). The nanoparticles form loose aggregates that can be up to 17 μm in size (Morefield et al., 2005). The surface area of AH is extremely large, estimated at 510 m^2^/g (Johnston et al., 2002). The point-of-zero charge (PZC) of AH is 11.4 giving the surface a positive charge at neutral pH. The other commercially available aluminum adjuvant is AP, which is chemically aluminum hydroxyphosphate, Al(OH)*_x_*(PO_4_)*_y_*. The ratio of surface hydroxyls and phosphate varies depending on the manufacturing conditions resulting in a PZC that varies between 4.5 and 5.5 and a negative charge at neutral pH. In contrast to the crystalline structure of AH, AP is amorphous by X-ray diffraction analysis (Burrell et al., 2000a). The primary AP particles are plate-like, 50 nm in diameter, and form loose aggregates that are approximately 3 μm in size (Burrell et al., 2000b; Morefield et al., 2005). The opposite surface charges of AH and AP in the pH range used for vaccine formulations affect the electrostatic interactions between the adjuvants and vaccine antigens as discussed in more detail below. Another difference between AH and AP is that AP more readily dissolves following injection. This was demonstrated *in vivo* with ^26^Al-labeled AH and AP injected intramuscularly into rabbits. Analysis of blood and tissue samples over a 28 day period revealed that about three times as much ^26^Al was released from AP than from AH (Flarend et al., 1997).

Another commercially available aluminum-containing adjuvant is Imject^®^ Alum. It is commonly used for experimental and basic immunological studies, but it is chemically very different from the AH and AP adjuvants that are used in human and veterinary vaccines. Imject^®^ Alum consists of crystalline magnesium hydroxide (40 mg/mL) and amorphous aluminum hydroxycarbonate (40 mg/mL; Hem et al., 2007). The composition of the aluminum component is different from AH and AP, while magnesium by itself has effects on the immune system and inflammatory response (Exley et al., 2010). Magnesium ions inhibit macrophage activation by blocking certain calcium channels and this contributes to the anti-inflammatory effect of magnesium (Lee et al., 2011). While Imject^®^ Alum clearly has adjuvant activity, it should not be used in experiments aimed at elucidating the mechanism of aluminum adjuvants in licensed human vaccines.

## Immunostimulation by Aluminum-Containing Adjuvants

Highly purified vaccine antigens are usually poorly immunogenic because of insufficient stimulation of the innate immune system. Aluminum adjuvants are included in vaccines to enhance the immune response to purified viral and bacterial antigens such as hepatitis B surface antigen (HBsAg), human papilloma virus capsid proteins and inactivated bacterial toxins. Their incorporation in vaccine formulations increases the concentration and avidity of antigen-specific antibodies (Petty and Steward, 1977; Lefeber et al., 2003). The level of immunity that is obtained by vaccination varies markedly among individuals due to genetically determined polymorphisms in molecules that play a role in the immune response, stochastic variation in the repertoire of antigen-specific receptors, and non-genetic factors such as age, nutritional status, and environmental influences. Adjuvants increase the proportion of the vaccinated population that develops a protective immune response and the duration of the immune response, and may allow for fewer immunizations and a reduced amount of antigen per dose (Coffman et al., 2010). Adjuvants also shape the type of immune response that develops in response to the antigens included in the vaccine. Aluminum adjuvants primarily enhance antibody production and have little effect on the cell-mediated arm of the immune response. Recent studies have begun to shed light on the complex mechanisms that appear to underlie the immunostimulatory effect of aluminum adjuvants. In reviewing these studies, one should keep in mind that many experiments were carried out using intraperitoneal injections of Imject^®^ Alum in a few inbred strains of mice. Although elegant and informative in terms of the biology, the relevance to the mechanisms involved in the immune response to aluminum adjuvants injected intramuscularly in human beings remains to be determined.

## The Immune Response to Aluminum-Adjuvanted Vaccines

Immunization with aluminum-adjuvanted vaccines induces antibody-mediated protection directed by CD4 T cells. Naïve CD4 T cells differentiate into effector cells with specific functions based on molecular signals provided by dendritic cells and the local microenvironment in which the differentiation takes place. There is considerable plasticity among effector CD4 T cells, but they can be divided into subpopulations based on secretion of particular combinations of cytokines and on expression of distinct surface markers and transcription factors (Zhu et al., 2010; Okoye and Wilson, 2011). The effector CD4 T cells were initially divided into Th1 and Th2 cells (Mosmann et al., 1986). Th1 cells are characterized by the secretion of IFN-γ and expression of the transcription factor T-bet, and Th2 cells by the secretion of IL-4, IL-5, and IL-13, and the expression of GATA-3 (Zhu et al., 2010). Around 2003, two new subpopulations of CD4 effector T cells were recognized, namely Th17 cells which secrete the cytokines IL-17A and IL-22 and express RORγt, and inducible regulatory T cells (iTreg) that express Foxp3 (Zhu et al., 2010). More recently, follicular T helper cells (T_FH_) were identified as a separate CD4 T cell subpopulation based on the expression of CXCR5, PD1, and the transcription factor BCL-6 (Johnston et al., 2009; Nurieva et al., 2009; Yu et al., 2009). These studies suggested that T_FH_ rather than Th2 cells are critical for antibody-mediated immune responses. Earlier reports showed that aluminum adjuvants direct differentiation of CD4 T cells to Th2 effector cells *in vivo* and *in vitro* and do not support the differentiation of Th1 cells (Brewer et al., 1996; Comoy et al., 1997; Cunningham et al., 2004; Sokolovska et al., 2007). The ability of aluminum adjuvants to induce a Th2-biased immune response is the basis of the common use of these adjuvants in the induction of allergic diseases in mouse models. More recent studies showed that injection of mice with alum-precipitated protein induced a marked increase of antigen-specific T_FH_ cells in the draining lymph nodes (Serre et al., 2011a,b). Comparison of T_FH_ and non-T_FH_ CD4 T cells suggested that T_FH_ cells rather than Th2 cells were the major subpopulation of IL-4-secreting CD4 T cells (Serre et al., 2011a). Taken together these older and more recent studies suggest that aluminum adjuvants induce the differentiation of Th2 cells that drive an eosinophilic inflammatory response and T_FH_ cells that stimulate antibody production. In addition aluminum-adjuvanted vaccines may support the differentiation of Th17 cells in mice. These cells are thought to be important in the protection against extracellular bacterial and fungal infections. A vaccine containing a yeast-expressed recombinant protein with AH induced protection against *Staphylococcus aureus* and *Candida albicans*. Protection was dependent on IL-17A and induction of Th17 cells in draining lymph nodes was demonstrated (Lin et al., 2009). A whole cell *Streptococcus pneumoniae* vaccine formulated with AH induced a robust IL-17A response in mice (HogenEsch et al., 2011). This response was not observed with AP or in the absence of AH. Earlier work had indicated that IL-17A is critical for protection against colonization following vaccination (Lu et al., 2008). These two examples of AH-supported Th17 induction used either a very high dose (300 μg) of a highly mannosylated protein or a poorly defined mixture of antigens. It remains to be seen if aluminum adjuvants support Th17 differentiation when combined with more conventional protein antigens. Interestingly, the induction of Th17 differentiation can be driven by IL-1 and IL-18 (Conforti-Andreoni et al., 2011), two cytokines that are induced by aluminum adjuvants as discussed in more detail below.

Evidence for a Th2-biased immune response in people is less convincing and the few available studies suggest a response driven by a mixed population of Th1, Th2, and possibly other CD4 effector cells. Peripheral blood mononuclear cells (PBMC) isolated from individuals immunized with keyhole limpet hemocyanin (KLH) combined with AH secreted mostly IL-5, IL-10, and IL-13 upon restimulation *in vitro* and induced IgG1 and IgG4 anti-KLH antibodies consistent with a Th2 response (Spazierer et al., 2009). However, restimulation of PBMC from the same volunteers with tetanus toxoid induced IFN-γ secretion and no IL-13 suggestive of a Th1 response. Although the vaccination and natural exposure histories were not specified, most tetanus vaccines contain aluminum adjuvants suggesting that aluminum-adjuvanted tetanus vaccines stimulated a Th1-biased response. Aluminum adjuvants are also used in immunotherapy of autoimmune and allergic diseases. Two subcutaneous injections of the 65 kDa isoform of glutamic acid dehydrogenase 65 (GAD65), an autoantigen in type 1 diabetes mellitus, formulated with AH (0.5 mg aluminum/dose) induced an antibody and T cell response. The T cell response was mixed with a significant increase of *ex vivo* secretion of IFN-γ, IL-17, IL-10, and type 2 cytokines, and development of FoxP3+, CD25+ CD4 T cells (Ludvigsson et al., 2008; Hjorth et al., 2011). A phase II clinical trial of GAD65 with AH failed to show efficacy in halting or delaying the development of diabetes mellitus (Wherrett et al., 2011). Preparations of allergens with aluminum hydroxide adjuvant are widely used for subcutaneous desensitization of patients with allergies (Francis and Durham, 2004). Possible explanations for the effect of this treatment include a shift away from Th2 responses and induction of IgE-blocking IgG antibodies and regulatory T cells (Eifan et al., 2011). *In vitro* exposure of PBMC from allergic individuals to allergen and aluminum hydroxide adjuvant reduced the secretion of IL-5 and IL-13 in comparison with allergen only, while the adjuvant had little effect on the secretion of IL-10, IL-12, and IFN-γ (Wilcock et al., 2004).

Proteins taken up by antigen-presenting cells are typically processed and peptides presented via MHC II molecules to CD4 T cells. In some cases, peptides from extracellular proteins can enter the MHC I presentation pathway and be presented to CD8 T cells. Dendritic cells are most efficient among antigen-presenting cells in cross-presentation of antigens (Joffre et al., 2012). The mechanisms involved in cross-presentation are not completely understood and vary depending on the antigen and delivery system (Compeer et al., 2012; Joffre et al., 2012). Immunization of mice with aluminum-adjuvanted vaccines does not effectively prime the development of cytotoxic T cells (CTLs; Dillon et al., 1992; Kwissa et al., 2003; Garulli et al., 2008). However, following immunization of ovalbumin (OVA) with AH or alum-precipitated OVA, OVA-specific CD8 T cells are activated and proliferate. The activated T cells express IFN-γ, but fail to differentiate into CTLs (McKee et al., 2009; Serre et al., 2010; MacLeod et al., 2011). Cross-presentation of OVA was dependent on CD8+ dendritic cells, a subset of DCs specialized in cross-presentation (MacLeod et al., 2011). The addition of a TLR4 agonist, monophosphoryl lipid A (MPLA), was required for induction of CTL differentiation (MacLeod et al., 2011). MPLA induces the secretion of IL-12 which is necessary for the terminal differentiation of CD8 T cells (Curtsinger et al., 2003; Pearce and Shen, 2007).

## Role of Adsorption in the Immunostimulating Effect of Aluminum Adjuvants

**Figure 1:**
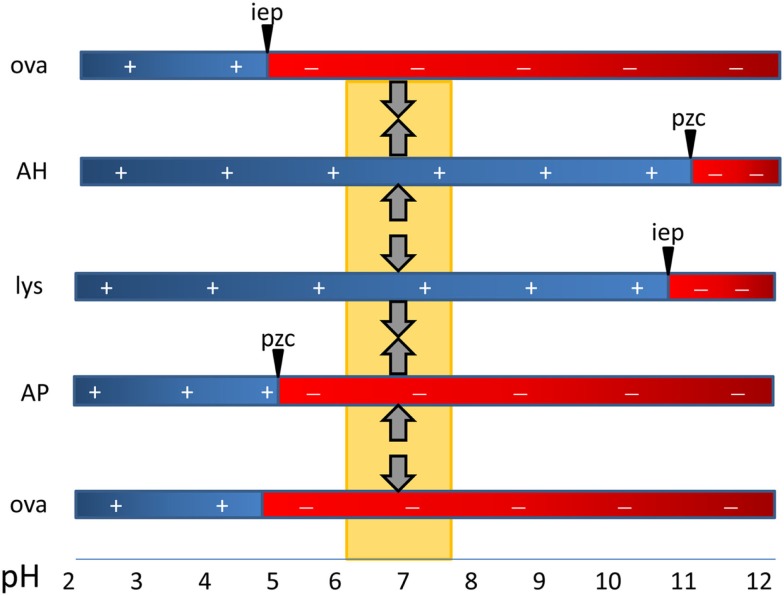
**The critical role of the type of aluminum adjuvant and pH in the adsorption of vaccine antigens via electrostatic mechanisms**. The point-of-zero-charge (PZC) of aluminum hydroxide adjuvant (AH) is 11.4 and that of aluminum phosphate adjuvant (AP) is approximately 5. At the pH of vaccine formulations (about 6–7.5), AH has a positively charged surface and attracts negatively charged proteins such as ovalbumin with a isoelectric point of 4.6. The surface of AP is negatively charged and it repels similarly charged proteins (OVA) and binds proteins with a high i.e.p. such as hen egg lysozyme (lys).

Antigens adsorb to aluminum adjuvants via hydrophobic and van der Waals forces, via electrostatic attraction and by ligand exchange (Hem and HogenEsch, 2007). Electrostatic attraction occurs when antigen and the adjuvant surface have opposite charges. The surface of AH is positively charged at neutral pH and attracts antigens with an isoelectric point (i.e.p.) less than 7, whereas AP is negatively charged and attracts proteins with an i.e.p. greater than 7 (Figure 1). Fusion proteins can consist of peptides with opposite i.e.p.’s resulting in a bipolar molecule. This likely affects the orientation of the antigens on the adjuvant surface when adsorbed to AH vs. AP, but no effect on the immune response was observed (Dagouassat et al., 2001). Antigens adsorbed via electrostatic mechanisms are quickly released upon exposure to interstitial fluid (Iyer et al., 2003; de Veer et al., 2010). Ligand exchange is the strongest attractive force between antigens and aluminum adjuvants. Aluminum has a higher affinity for phosphate than for hydroxyls and phosphates will displace surface hydroxyls on aluminum adjuvants. Molecules with multiple terminal phosphate groups have a very high affinity for AH as they adsorb via ligand exchange. The affinity can be modulated by pre-treatment of AH with phosphate buffer resulting in replacement of hydroxyls by phosphates. Antigens adsorbed via ligand exchange mechanism are slowly released from the adjuvant following exposure to interstitial fluid.

Adsorption is generally thought to be important for the immunostimulatory effect of aluminum adjuvants (Gupta, 1998). The particulate nature of adsorbed antigens facilitates uptake by antigen-presenting cells via phagocytosis (Mannhalter et al., 1985; Morefield et al., 2005). Adsorbed antigens are more slowly released from the injection site (Leeling et al., 1979; Weissburg et al., 1995; Noe et al., 2010), but the kinetics are highly dependent on the strength of the adsorption. Electrostatically adsorbed antigens are rapidly released and diffuse away from the injection site at a higher rate than antigens adsorbed via ligand exchange (Noe et al., 2010). The retention of antigens at the injection site allows time for inflammatory cells and antigen-presenting cells to accumulate at the injection site and to interact with vaccine antigens. This is consistent with early experiments by Holt who demonstrated that removal of the injection site within 4 days after subcutaneous injection of guinea pigs with diphtheria toxoid adsorbed to AP interfered with the development of an antibody response. In contrast, removal of the injected ear 2 h after subcutaneous injection of AH with antigen did not affect the magnitude or duration of the immune response (Hutchison et al., 2012). This would suggest that neither adsorption nor the local inflammatory response at the injection site is necessary for the immunostimulatory effect of aluminum adjuvants. It is difficult to reconcile this with the studies by Holt. Unfortunately, the results of ear ablation were not reported following injection of antigen only. It is possible that tissue trauma resulting from resection of the ear induced sufficient inflammatory signals to stimulate the development of the immune response.

Aluminum adjuvants can also stimulate the immune response to non-adsorbed antigens (Berthold et al., 2005; Romero Mendez et al., 2007). The adjuvant and antigen need to be injected at the same site in order for the enhanced immunogenicity to occur (Chang et al., 2001; Eisenbarth et al., 2008). The need for adsorption appears to decrease with larger antigen doses (Berthold et al., 2005). This is probably caused by interactions between the antigen and the extracellular matrix resulting in retention of a portion of the injected antigen. This is indicated by the kinetics of subcutaneously injected soluble protein (Noe et al., 2010) and further supported by pharmacokinetic studies with subcutaneously injected monoclonal antibodies showing significant retention of protein at the site of injection (Joshi et al., 2006; Xu et al., 2010). *In vitro* experiments suggests that a variable but significant portion of monoclonal antibodies is retained at the injection site via electrostatic interactions with the extracellular matrix (Mach et al., 2011). This is also likely to happen with certain vaccine antigens. When higher doses of non-adsorbed antigens are used, a sufficient amount of antigen is retained at the injection site for interaction with inflammatory cells recruited in response to the co-injected aluminum adjuvants. On the other hand, too tight adsorption has a negative effect on the immunogenicity of the vaccine as demonstrated most clearly with alpha-casein, a small 24 kDa protein with eight phosphate groups allowing for extensive ligand exchange interactions with AH (Hansen et al., 2007). The adsorptive strength (affinity) of alpha-casein for AH could be reduced by pre-treatment of AH with phosphate buffer and this resulted in an increased antibody and T cell response (Hansen et al., 2007). High adsorptive strength probably interferes with antigen processing and prevents T cell activation. Similarly, the adsorptive strength of HBsAg which is embedded in a phospholipid membrane can be reduced by pre-treatment of AH with phosphate. The formulation of HBsAg with phosphate-treated AH or AP induced a greater antibody response than HBsAg with AH (Kwissa et al., 2003; Hansen et al., 2009).

While adsorption to aluminum adjuvants usually enhances the immune response to antigens, it can affect the physical and chemical stability of antigens over time (Clapp et al., 2011). Proteins are subjected to various degradation processes including deamidation, oxidation, and hydrolysis that are dependent on pH and temperature (Manning et al., 2010). The pH of the microenvironment of the charged aluminum adjuvant surface is different from the bulk formulation pH because of attraction of ions from the solution. The positively charged surface of AH attracts negatively charged hydroxyl ions increasing the microenvironment pH by as much as two units (Wittayanukulluk et al., 2004). This change in pH can accelerate the degradation of adsorbed antigens. Proteins adsorbed onto solid surface also tend to unfold causing a loss of the secondary and tertiary structure (Manning et al., 2010). This often results in exposure of hydrophobic parts of the proteins and an increase of hydrophobic interactions with aluminum adjuvants. This likely contributes to reduced desorption of antigens from aluminum adjuvants in aged vaccines (Shi et al., 2001a; Vessely et al., 2009). Several biophysical assays including differential scanning calorimetry, intrinsic (tryptophan) and extrinsic fluorescence, and Fourier transform infrared spectroscopy, have been successfully employed in recent years to assess the conformational stability of proteins adsorbed onto aluminum adjuvants (Jones et al., 2005; Peek et al., 2007; Ausar et al., 2011; Iyer et al., 2012; Ljutic et al., 2012). Adsorption usually decreases the stability of proteins, but increased stability of AH-adsorbed proteins was observed in a few cases (Estey et al., 2009; Iyer et al., 2012). Few studies have directly examined the effect of the adsorption-induced structural changes on the immune response to the vaccine. Studies of freshly prepared AH-adsorbed recombinant protective antigen (rPA) of *Bacillus anthracis* (rPA) identified minimal changes in the protein structure (Soliakov et al., 2012). However, others observed marked structural changes in rPA upon aging the vaccine for 3 weeks, and this was associated with a decrease of neutralizing antibodies in mice injected with the aged formulation compared with the freshly prepared vaccine (Wagner et al., 2012). Adsorption-induced changes in the protein structure can be reduced by formulation of the vaccine with a phosphate buffer which alters the microenvironment pH and may reduce the adsorptive strength (Wittayanukulluk et al., 2004; Iyer et al., 2012). An alternative strategy is to add stabilizers such as sucrose, sorbitol and trehalose to the vaccine formulation (Peek et al., 2007; Iyer et al., 2012). These compounds stabilize proteins in solution and adsorbed onto aluminum adjuvants, but their addition to vaccine formulation scan decrease the adsorption of protein antigens (Peek et al., 2007; Vessely et al., 2007).

## Role of Inflammation in the Immunostimulating Effect of Aluminum Adjuvants

Inflammation induced at the injection site is thought to be important for the adjuvant effect of AH and AP. Intramuscular injection of mice and guinea pigs with tetanus toxoid vaccines with AH resulted in necrosis of muscle fibers and inflammation with edema and infiltration of leukocytes (Goto and Akama, 1982; Goto et al., 1997). This is associated with increased expression of mRNAs for chemokines, cytokines, and cell adhesion molecules (Mosca et al., 2008). The majority of leukocytes were neutrophils during the first 72 h, followed by accumulation of increasing numbers of macrophages at 1 week after injection (Goto and Akama, 1982). The macrophages form granulomas that can persist for months (Goto and Akama, 1982; Verdier et al., 2005). Although these granulomas contain aluminum adjuvant and antigen, their presence is not required for the immune response as removal of the injection site after 7 days did not interfere with the magnitude or duration of the immune response (Holt, 1950). Goto and Akama (1982) found few eosinophils at the injection site in contrast to an earlier report that approximately 25% of the inflammatory cells were eosinophils 4 days following intramuscular injection of tetanus toxoid with AH or AP (Walls, 1977). In a more recent study, the composition of the inflammatory cells following intramuscular injection of mice with OVA and AH was analyzed by flow cytometry at 24 h after injection (Calabro et al., 2011). The majority of infiltrating cells were neutrophils and inflammatory monocytes with few eosinophils (Calabro et al., 2011). Other studies examined the kinetics of the inflammatory response following intraperitoneal injection of aluminum-containing adjuvants. This route of injection allows for recovery of cells without the need of enzymatic treatment and for quantification of the concentration of cytokines in the peritoneal fluid, but of course is different from the normal route of injection of vaccines. Injection of aluminum-containing adjuvant with or without protein antigens such as OVA resulted in rapid secretion of chemokines and cytokines into the peritoneal fluid (Kool et al., 2008b; McKee et al., 2009; Korsholm et al., 2010). An increase of neutrophils occurred as early as 6 h after injection (Kool et al., 2008b; Korsholm et al., 2010). This was followed by an increased number of inflammatory monocytes and myeloid and plasmacytoid dendritic cells. The number of eosinophils was increased at 24 h after injection (Walls, 1977; Kool et al., 2008b; McKee et al., 2008, 2009; Korsholm et al., 2010), and continued to increase reaching a peak at 4–8 days (Walls, 1977). The 24-h accumulation of eosinophils was partially dependent on mast cell-derived IL-5 and histamine and unidentified factors secreted by macrophages (McKee et al., 2009). The increase of eosinophils in response to AH was diminished in thymectomized mice suggesting a role for T cells (Walls, 1977). Neutrophils orchestrate the recruitment of inflammatory monocytes via the release of granule proteins such as azurocidin and LL-37 (Soehnlein and Lindbom, 2010). In spite of this role, depletion of neutrophils with monoclonal antibodies prior to immunization with lysozyme and aluminum adjuvant increased activation of antigen-specific T cells and the antibody response (Yang et al., 2010). The neutrophils appeared to compete with antigen-presenting cells for antigen and to interfere directly with antigen presentation (Yang et al., 2010). Inflammatory monocytes recruited to the injection site can differentiate into macrophages and dendritic cells. They take up antigen and transport it to the draining lymph node while undergoing differentiation into dendritic cells. This is consistent with *in vitro* experiments which show that aluminum adjuvants induce differentiation of human monocytes to cells with phenotypic and functional characteristics of dendritic cells (Ulanova et al., 2001; Rimaniol et al., 2004; Seubert et al., 2008). Depletion of peritoneal macrophages with clodronate-containing liposomes did not affect the immune response to intraperitoneally injected OVA with aluminum adjuvant (McKee et al., 2009). However, depletion of dendritic cells nearly completely abolished T cell responses and antibody production indicating a critical role for dendritic cells in the immune response to aluminum-adjuvanted vaccines (Kool et al., 2008b). Intraperitoneal injection of aluminum adjuvant also induced an increase of Gr-1+, IL-4+ cells in the spleen, most of which are eosinophils (Jordan et al., 2004; Wang and Weller, 2008). Although these eosinophils appeared to enhance B cell priming and early IgM antibody production, the absence of eosinophils did not affect the quality or magnitude of the antibody response to aluminum-adjuvanted vaccines (McKee et al., 2009). Recently, basophils were reported to serve as antigen-presenting cells for Th2-biased immune responses (Perrigoue et al., 2009; Sokol et al., 2009; Yoshimoto et al., 2009). These reports were based on depletion of basophils with anti-FcεRI (Mar-1) antibodies. It was subsequently shown that mice have a subset of FcεRI+ dendritic cells that are depleted by Mar-1 antibody treatment in addition to basophils (Hammad et al., 2010). Genetically engineered mice that lack basophils are capable of mounting a normal antibody response to aluminum-adjuvanted vaccines indicating that basophils are not necessary (Ohnmacht et al., 2010). Thus, among the different cells recruited to the vaccine injection site, the recruitment of inflammatory monocytes and subsequent differentiation into dendritic cells appears to be critical for the development of the immune response to aluminum-adjuvanted vaccines.

## Aluminum Adjuvants and Dendritic Cells

*In vitro* studies with mouse bone marrow-derived dendritic cells have demonstrated that aluminum adjuvants have a direct effect on dendritic cells. They increase the uptake of antigens and the presentation and activation of antigen-specific T cells (Morefield et al., 2005; Sokolovska et al., 2007; Ghimire et al., 2012). Both AH and AP induce secretion of IL-1β and IL-18 by dendritic cells (Li et al., 2007; Sokolovska et al., 2007). This also occurred in MyD88-deficient dendritic cells consistent with observations that immunostimulation by aluminum adjuvants *in vivo* was independent of signaling through Toll-like receptors (Schnare et al., 2001; Gavin et al., 2006). Dendritic cells incubated with aluminum adjuvants and OVA induced differentiation of naïve OVA-specific CD4 T cells to Th2 cells *in vitro*. This effect was blocked by antibodies against IL-1β and IL-18 suggesting a role for these cytokines in Th2 differentiation (Sokolovska et al., 2007). IL-1β and IL-18 are synthesized by cells as inactive cytoplasmic precursors that require cleavage by the enzyme caspase-1 in order to be released from the cells. Experiments with caspase-1 inhibitors demonstrated that aluminum adjuvants induce activation of caspase-1 resulting in the secretion of the active forms of IL-1β and IL-18 (Li et al., 2007; Sokolovska et al., 2007). Subsequent work showed that the secretion of IL-1βand IL-18 by dendritic cells incubated with aluminum adjuvants depends on the presence of the NOD-like receptor NLRP3 (Eisenbarth et al., 2008; Franchi and Nunez, 2008; Hornung et al., 2008; Kool et al., 2008a; Li et al., 2008). NLRP3 is a member of a family of cytoplasmic pattern recognition receptors and is activated in response to a variety of sterile stimuli such as ATP, uric acid crystals, silica, asbestos, and aluminum adjuvants (Martinon et al., 2009). Upon activation, NLRP3 forms a multimeric protein complex termed inflammasome that contains the adaptor protein apoptosis-associated speck-like protein (ASC) and the inactive precursor of caspase-1, procaspase-1. The close proximity of procaspase-1 molecules leads to autoproteolytic processing into active caspase-1 that in turn cleaves pro-IL-1β and pro-IL-18 into their active and secreted forms (Martinon et al., 2009). Activation of NLRP3 by aluminum adjuvants required phagocytosis of aluminum particles, acidification of lysosomes, and maturation of cathepsin B (Eisenbarth et al., 2008; Hornung et al., 2008). The aluminum particles caused disruption of the phagolysosomes and release of active cathepsin B into the cytoplasm which may be a sufficient signal for NLPR3 activation (Hornung et al., 2008). Phagocytosis and activation of cathepsin B in dendritic cells and macrophages can also lead to discharge of ATP via connexin and pannexin channels into the extracellular environment (Riteau et al., 2012). The extracellular ATP in turn binds to purinergic receptors including the P2X7 receptor which induces inflammasome activation. The *in vivo* relevance of caspase-1 and NLRP3-dependent secretion of IL-1β and IL-18 in the immunostimulatory effect of aluminum adjuvants is controversial. Experiments with NLRP3-deficient mice have demonstrated a marked suppression of antigen-specific IgE and IgG1 production (Eisenbarth et al., 2008; Li et al., 2008), suppression of IgE, but not IgG1 (Kool et al., 2008a), or no effect on antibody production at all (Franchi and Nunez, 2008; McKee et al., 2009). It is likely that these divergent results reflect differences in immunization protocols, type and amount of aluminum adjuvants, and mouse strains. Aluminum adjuvants also induce the secretion of IL-1α by dendritic cells in a caspase-1-dependent manner (Sharp et al., 2009). IL-1α and IL-1β bind to the same IL-1 receptor and have overlapping functions. However, the secretion of IL-1α was only partially dependent on phagocytosis and NLRP3 suggesting that a different pathway is involved in the secretion of this cytokine (Sharp et al., 2009). IL-1α, IL-1β, and IL-18 signaling requires the adaptor protein MyD88 (Adachi et al., 1998). The adjuvant effect of aluminum adjuvants *in vivo* is independent of MyD88 suggesting a limited role for these cytokines in immunostimulation by AH and AP (Schnare et al., 2001; Gavin et al., 2006).

Another possible mechanism of dendritic cell activation by aluminum adjuvants is binding to plasma membrane lipids. This led to reassortment of lipids and aggregation of lipid rafts resulting in activation of the syk kinase and phosphoinositide-3 kinase (PI3K) pathways (Flach et al., 2011). The lipid sorting resulted in abortive phagocytosis and uptake of antigen, but not the aluminum adjuvant. The authors suggested that the uptake of antigen via endocytosis without adjuvant prevents cross-presentation and would explain the ability of aluminum adjuvants to induce a humoral and CD4 T cell response, but not a CTL response. However, as discussed above, proteins combined with aluminum adjuvants can in fact activate CD8 T cells (Kwissa et al., 2003; Serre et al., 2010; MacLeod et al., 2011). Moreover, there is abundant evidence of intracellular localization of aluminum in macrophages and dendritic cells, both *in vitro* and *in vivo* (Rimaniol et al., 2004; Morefield et al., 2005, 2008). Activation of PI3K also occurs following binding of ATP to the P2X7 receptor and is required for inflammasome activation by extracellular ATP (Cruz et al., 2007). Inhibition of PI3K partially inhibited the secretion of IL-1β by dendritic cells (Mori et al., 2012).

Incubation of lipopolysaccharide (LPS)-activated macrophages with aluminum adjuvants induced secretion of prostaglandin E2 (PGE2; Kuroda et al., 2011). Damage of phagolysosomes by phagocytized aluminum particles caused activation of Syk and p38 MAP kinase pathways leading to the synthesis of PGE2. Prostaglandin E synthase-deficient mice immunized with OVA and aluminum adjuvant had decreased production of OVA-specific IgE, whereas the production of IgG1 and IgG2c were similar to wild-type mice.

In addition to direct effects on dendritic cells, aluminum adjuvants can also have indirect effects via endogenous activators. Cell injury and necrosis cause the release of intracellular molecules that activate innate immune cells. These molecules have been termed alarmins or danger-associated molecular patterns, and include High Mobility Group Box 1 (HMGB1), uric acid, ATP, heat shock proteins, DNA, IL-1α, and filamentous actin (Rock et al., 2010; Ahrens et al., 2012; Zhang et al., 2012). Several recent studies have suggested that uric acid, DNA, ATP, and HSP70 are involved in the immunostimulation by aluminum adjuvants (Kool et al., 2008b; Marichal et al., 2011; Riteau et al., 2012; Wang et al., 2012). Intraperitoneal injection of aluminum-containing adjuvant induced an increase of uric acid, and treatment of mice with uricase decreased activation of antigen-specific T cells in the draining lymph node suggesting a role for uric acid in the activation of the immune response by aluminum-containing adjuvants (Kool et al., 2008b). Aluminum adjuvants are commonly used for sensitization of mice in the establishment of mouse models of allergic airway disease. The induction of allergic airway disease was impaired upon treatment with uricase implying a role of uric acid in the development of the aluminum adjuvant induced allergic immune response (Kool et al., 2011). However, as discussed above, aluminum adjuvants induce a weak cytotoxic T cell response by themselves, whereas uric acid is a potent inducer of CTLs (Shi et al., 2003). Moreover, the role of uric acid following intramuscular injection of aluminum adjuvants remains to be determined. Both intraperitoneal and intramuscular injection of aluminum adjuvants with OVA induced cell death and release of DNA (Marichal et al., 2011). OVA-specific secretion of IgE and IgG1 following intraperitoneal injection was inhibited by treatment with DNAse around the time of injection. Host cell DNA itself acted as an adjuvant, and activated Th2 immune responses via the transcription factor interferon regulatory factor 3 (IRF3). Injection of IRF3-deficient mice with aluminum adjuvant induced similar accumulation of inflammatory cells in the peritoneal cavity and OVA-specific IgG1 as wild-type mice, but IRF3-deficiency abolished the OVA-specific IgE response (Marichal et al., 2011), suggesting a dissociation of the IgG1 and IgE response. Subcutaneous injection of aluminum-adjuvanted-OVA induced increased expression of membrane HSP70 in splenic dendritic cells (Wang et al., 2012). A similar effect was seen upon administration of OVA with known stress-inducing compounds. Inhibition of the function of HSP70 with a small molecule inhibitor in mice only decreased the IgG2a response which suggests that HSP70 is primarily involved with the induction of the weak Th1-component of the immune response to aluminum-adjuvanted vaccines in mice. This is consistent with the induction of predominant Th1 responses with HSP70 (Wang et al., 2002). Furthermore, HSP70 is a ligand of TLR4 (Vabulas et al., 2002), whereas the antibody response to aluminum-adjuvanted vaccines is independent of TLRs (Schnare et al., 2001; Gavin et al., 2006). This suggests a minimal role of HSP70 in the immunostimulation by aluminum adjuvants.

**Figure 2:**
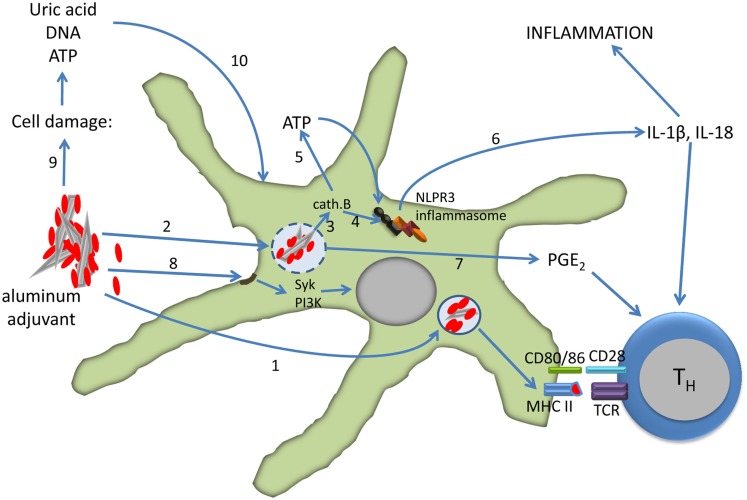
**Effect of aluminum adjuvants on dendritic cells**. The particulate nature of adsorbed protein (red) antigens facilitates phagocytosis and antigen presentation (1). Uptake of aluminum adjuvants may lead to destabilization and rupture of the phagolysosome (2) which results in activation of cathepsin B (3). Cathepsin B induces the assembly of the NLPR3 inflammasome directly (4) or via extracellular release of ATP through connexin and pannexin channels (5). Caspase-1, a component of the inflammasome, cleaves pro-IL-1β and IL-18 into active forms that are released from the cell (6). Phagolysosome destabilization also induces the secretion of PGE2 (7), and PGE2, IL-1β, and IL-18 all support the differentiation of CD4 T cells into Th2 cells. Aluminum adjuvants also directly interact with the lipid rafts in the cell membrane which results in activation of the Syk and PI3-kinase signaling pathways (8). Injection of aluminum-adjuvanted vaccines causes cell damage and necrosis with release of uric acid, ATP, and DNA (9). These molecules in turn activate dendritic cells (10). See the text for more details and references.

In summary, the formulation of vaccines with aluminum adjuvants activate dendritic cells both directly and indirectly leading to the differentiation of CD4 T cells into effector cells (Figure 2). The relative roles of the direct and indirect mechanisms depend on several factors, including the dose of aluminum in the vaccine, dose volume, and route of injection.

## Aluminum in Combination Adjuvants

A weakness of aluminum adjuvants is the failure to induce a robust cell-mediated immune response which limits its utility in vaccines for diseases in which such an immune response is protective. The addition of other immunostimulatory molecules to vaccines can potentially overcome this limitation. The goal of such combination adjuvants is to develop the “perfect mix” to achieve the desired type and magnitude of an immune response (Guy, 2007). When aluminum adjuvants are combined with other immunostimulatory molecules, the interactions are subject to the same mechanisms that are involved with adsorption of antigens. The adsorbed molecules are retained at the injection site which can prevent potential systemic toxicity while increasing the interactions with newly recruited antigen-presenting cells. However, too tight adsorption through ligand exchange interferes with the activity of the immunostimulatory molecules such as LPS and CpG oligonucleotides (ODN; Shi et al., 2001b; Aebig et al., 2007). Adsorption of immunostimulatory molecules to aluminum adjuvants can also affect the adsorption of antigens (Aebig et al., 2007). Thorough optimization of the formulation conditions is necessary to achieve the most effective vaccine combination.

As aluminum adjuvants do not activate TLRs, the addition of TLR agonists is a logical choice. A combination adjuvant composed of AH and the TLR4 agonist MPLA is used in licensed vaccines against hepatitis B and human papilloma virus (Garcon, 2010). MPLA is a derivative of LPS with greatly reduced toxicity. LPS has two phosphates which allow binding with high affinity to AH via ligand exchange and this neutralizes the activity of LPS (Shi et al., 2001b). As the name implies, MPLA has one phosphate which reduces the affinity for AH and enhances the adjuvant effect of AH. The combination of AH with MPLA induces a stronger antibody response and a shift from Th2 to Th1 cytokine production in spleen cells (Giannini et al., 2006; Didierlaurent et al., 2009). As mentioned earlier, the addition of MPLA to AH also enables differentiation of activated CD8 T cells into CTLs (MacLeod et al., 2011). The effect of other TLR agonists on the immune response to aluminum-adjuvanted vaccines has been investigated, but this has not yet led to licensed products. The TLR9 agonist CpG ODN enhanced the antibody response in mice to hepatitis B antigen formulated with AH with a shift from IgG1 to IgG2a antibodies, and induced a CTL response (Davis et al., 1998). A similar enhancement has been reported in people injected with AH-adjuvanted hepatitis B vaccine with added CpG ODN (Cooper et al., 2004). The CpG ODN bind more strongly to AH than AP and the binding to AH is reduced in the presence of phosphate buffer suggesting that ligand exchange underlies the adsorption to AH (Aebig et al., 2007). Adsorption of CpG ODN is important for the enhancement of the immune response and excess non-adsorbed CpG ODN appears to inhibit the immune response (Mullen et al., 2007).

Muramyl dipeptide (MDP), a component of the mycobacterial cell wall, activates the innate immune system via NOD2, a cytoplasmic pattern recognition receptor. The combination of AH with MDP as adjuvant did not enhance the antibody response to a bacterial antigen in comparison with AH alone, but it stimulated a stronger IFN-γ response (Moschos et al., 2006). Quil A, a partially purified mixture of saponins from the *Quillaja saponaria* tree, activates NALP3 inflammasomes in dendritic cells similar to aluminum adjuvants (Li et al., 2008). In spite of the overlapping mechanism of dendritic cell activation, the combination of AH and Quil A enhanced the antibody response and CTL response to protein antigens in comparison with AH alone (Wu et al., 1992; Lofthouse et al., 1995). However, Quil A failed to enhance the immune response to a bacterial antigen when combined with AH in another study (Moschos et al., 2006).

## Safety of Aluminum-Containing Adjuvants

Aluminum-containing adjuvants have been used for more than 70 years in billions of doses of vaccines, and have an excellent safety record (Butler et al., 1969; Edelman, 1980; Jefferson et al., 2004). The maximum amount of aluminum adjuvant allowed in human vaccines in the US is 0.85 mg Al/dose, and the amount in licensed vaccines ranges from 0.125 to 0.85 mg Al/dose (Baylor et al., 2002). Aluminum is an abundant metal in the environment and is daily ingested in food and water (Willhite et al., 2012). Aluminum is also commonly used in antacids and antiperspirants. However, only small amounts of aluminum are absorbed via the intestinal barrier and the skin. Most of the aluminum is excreted via the kidneys. Aluminum toxicity from occupational exposure, renal disease, and parenteral nutrition is associated with neurologic disease and bone disease (Willhite et al., 2012). The pharmacokinetics of aluminum following intramuscular injection of AH and AP (0.85 mg/dose) was studied in rabbits using the rare ^26^Al isotope as a tracer (Flarend et al., 1997). The data indicated that 17% of AH and 51% of AP was released into the blood circulation over a 28 day period. Based on these and other data, it was recently estimated that the concentration of aluminum in blood derived from vaccines administered to infants during the first year of life remains well below the minimum risk level established by the Agency for Toxic Substances and Disease Registry (Mitkus et al., 2011).

As discussed above, injection of aluminum adjuvants induces a local inflammatory reaction, and this can be associated with clinical experiences of pain, swelling, and redness at the injection site. These localized reactions are usually mild and of short duration. In fact, aluminum adjuvants reduce the prevalence and severity of systemic adverse reactions by binding and slowly releasing molecules thereby reducing toxicity. This has been demonstrated for LPS which bind tightly to AH via ligand exchange involving the two terminal phosphate groups neutralizing the activity (Norimatsu et al., 1995; Shi et al., 2001b). The prevalence of systemic adverse effects was greater in children immunized with plain DTP vaccine than in those receiving DTP with AH (Butler et al., 1969). In allergen immunotherapy, subcutaneous injection of AH-adsorbed allergen induced fewer local reactions than the allergen in solution (Rueff et al., 2004).

The prevalence and severity of local inflammatory reactions is affected by the site of injection. Local reactions were greater following subcutaneous than intramuscular immunization of aluminum-adjuvanted DT and anthrax vaccines (Mark et al., 1999; Pittman et al., 2002). This can be attributed in part to the superficial location of subcutaneous reactions, but can also be the result of physiological differences between the tissues. Casein tightly adsorbed to AH persisted at the injection site longer following subcutaneous than intramuscular injection possibly reflecting differences in lymph flow rate between the subcutis and skeletal muscle (Noe et al., 2010). Contact hypersensitivity to aluminum is rare, but it has been reported following vaccination with aluminum-adjuvanted vaccines, hyposensitization with aluminum-adjuvanted allergens and the use of aluminum-containing antiperspirants (Bergfors et al., 2003; Netterlid et al., 2009; Garg et al., 2010). It manifests itself by persistent itching nodules and contact dermatitis to antiperspirants. The contact hypersensitivity response can contribute to the inflammatory reaction at the injection site.

A muscle disease termed macrophage myofasciitis characterized by muscle weakness, myalgia, and fever was attributed to vaccination with aluminum-adjuvanted vaccines (Gherardi et al., 1998). Biopsies of the deltoid muscle demonstrated accumulation of macrophages with intracellular accumulation of aluminum hydroxide. The patients had received vaccinations 3 months to several years prior to the biopsy. The light microscopic lesions are similar to those described at the injection site in people and experimental animals injected intramuscularly with aluminum adjuvants (Mrak, 1982; Verdier et al., 2005; Lach and Cupler, 2008). Such lesions have been demonstrated in non-human primates 12 months after injection (Verdier et al., 2005). There is no evidence that these localized injection site reactions are related to systemic muscle disease (Lach and Cupler, 2008).

In veterinary medicine, an increased prevalence of sarcomas in cats was reported in the early 1990s (Hendrick et al., 1992). The location, histologic features, and biological behavior suggested a distinct type of tumor associated with the site of vaccination (Hendrick et al., 1994; Doddy et al., 1996). The prevalence is estimated at about 1:3,000–1:10,000 vaccinations. Aluminum was identified in some of the sarcomas that were present at sites of previous vaccination in cats (Hendrick et al., 1992). This suggested a possible causal role of aluminum adjuvants in the development of sarcomas, but subsequent epidemiologic studies failed to demonstrate an association between particular types of vaccines and sarcomas (Kass et al., 2003). However, a recent report indicated that cats with sarcomas in the rear lower limb were less likely to have received recombinant rabies vaccine without adjuvant than an adjuvanted inactivated rabies vaccine (Srivastav et al., 2012). Non-adjuvanted vaccines induced less severe inflammation than adjuvanted vaccines at the subcutaneous injection site in cats (Day et al., 2007). Sarcomas have also been reported in cats in association with injections other than vaccines including long acting penicillin and corticosteroid medications (Kass et al., 2003; Srivastav et al., 2012). This suggests that there is a small subpopulation of cats that is prone to develop sarcomas at sites of chronic inflammation, and no specific role for aluminum adjuvant in the pathogenesis of these neoplasms. There is no evidence of sarcomas caused by injection of aluminum-adjuvanted vaccines or allergens in people (Jekel et al., 1978).

## Conclusion

Aluminum-containing adjuvants are widely used in preventive vaccines against infectious diseases and in immunotherapy of allergies. Recent studies have begun to increase our understanding of the mechanisms involved in adsorption of antigens onto aluminum adjuvants and the effect of adsorption on the stability of antigens and the immune response. Aluminum adjuvants appear to enhance the immune response via several molecular pathways, but more work is needed to understand the interactions and relative importance of these pathways. This knowledge will help to determine the optimal formulation conditions for effective and safe aluminum-adjuvanted vaccines.

## Conflict of Interest Statement

The author declares that the research was conducted in the absence of any commercial or financial relationships that could be construed as a potential conflict of interest.
